# ^210^Po and ^210^Pb in King Bolete (*Boletus edulis*) and Related Mushroom Species: Estimated Effective Radiation Dose and Geospatial Distribution in Central and Eastern Europe

**DOI:** 10.3390/ijerph18189573

**Published:** 2021-09-11

**Authors:** Dagmara Strumińska-Parulska, Aleksandra Moniakowska, Grzegorz Olszewski, Jerzy Falandysz

**Affiliations:** 1 Laboratory of Toxicology and Radiation Protection, Faculty of Chemistry, University of Gdańsk, 63 Wita Stwosza Street, 80-308 Gdańsk, Poland; aleksandra.moniakowska@ug.edu.pl (A.M.); grzegorz.olszewski@ug.edu.pl (G.O.); 2 Department of Health, Medicine and Caring Science, Division of Diagnostics and Specialist Medicine, Linköping University, 581 83 Linköping, Sweden; 3 Department of Toxicology, Faculty of Pharmacy, Medical University of Łódź, 1 Muszyńskiego Street, 90-151 Łódź, Poland; jerzy.falandysz@gmail.com

**Keywords:** food toxicology, foodstuffs, forest, polonium, lead, radiochemistry, trace elements

## Abstract

^210^Po and ^210^Pb occur naturally and are the most radiotoxic isotopes of the uranium (U) decay chain. Samples of *Boletus edulis* and related mushroom species, including *B. pinophilus*, *B. reticulatus*, *B. luridus* and *B. impolitus*, collected from Poland and Belarus were investigated for the activity concentrations of these isotopes and also for their potential health risk through adult human consumption. The results showed that spatially, the occurrence of ^210^Po and ^210^Po was heterogeneous, with activities varying from 0.91 to 4.47 Bq∙kg^−1^ dry biomass and from 0.82 to 5.82 Bq∙kg^−1^ db, respectively. Caps and stipes of the fruiting bodies showed similar levels of contamination. Consumption of boletes foraged in Poland could result in exposure to a combined radiation dose of 10 µSv∙kg^−1^ db from both isotopes. This dose is not significant compared to the total annual effective radiation dose of ^210^Po and ^210^Pb (54–471 µSv∙kg^−1^) from all sources, suggesting that these mushrooms are comparatively safe for human consumption.

## 1. Introduction

Dried mushrooms (carpophores of *Basidiomycota*) are relatively rich in mineral constituents (the water content of fresh mushrooms is around 90%), but radioactive elements can form a significant part of these and pose a health risk to consumers. A key example is the contamination of wild mushrooms with artificial nuclides of radiocaesium (^134/137^Cs) after nuclear cataclysms. These were the most commonly studied nuclides in mushrooms as documented and reviewed [1,2,3,4,5,6,7,8,9]. ^210^Po and its parent nuclide ^210^Pb originate from the ^238^U (uranium) decay chain. They are also the most toxic amongst the uranium chain radioactive elements, and their half-lives are 138.38 days for ^210^Po and 22.3 years for ^210^Pb [10,11,12]. ^210^Po, ^210^Pb, ^226^Ra and ^40^K as natural nuclides contribute mainly to the effective radiation background in biota that are unexposed to anthropogenic radioactivity: ^210^Po + ^210^Pb + ^226^Ra annually contributes 165 µSv to a daily diet, while ^40^K provides 140 µSv [13].

The family of the *Boletus* fungi is rich in genera and species that are cosmopolitan and collected worldwide [14,15]. The majority from the genus *Boletus* are edible (only a few are toxic, e.g., *Rubroboletus satanas*), tasty, and valued by local consumers. Perhaps the most remarkable and prized of these, the King Bolete, *Boletus edulis,* occurs relatively frequently and is native to forests of the temperate climate [14].

The objectives of this study were to evaluate the occurrence, distribution within fruiting bodies and possible risk (to consumers) from ^210^Po and ^210^Pb that tend to accumulate in *Boletus edulis, Boletus pinophilus, Boletus reticulatus, Boletus luridus* and *Boletus impolitus* mushrooms and also to prepare, based on the results, interpolation maps to spatially characterize the occurrence of both nuclides in boletes in Poland.

## 2. Materials and Methods

The bolete mushrooms studied, species such as *B. edulis, B. pinophilus, B. reticulatus, B. luridus* and *B. impolitus*, were collected from 25 woodlands/forested sites across Poland (the Pomerania, Kujawy, Warmia, Podlasie and Masuria regions, and the Tatra and Sudety Mts.). Also included in the study were *B. reticulatus* samples from two locations in Belarus (Gomel and Minsk regions) from our depository. To obtain an insight into the ^210^Po and ^210^Pb distribution within the fruitbody, some mushroom samples were separated into cap and stipe during preparation (samples/pools id. 1–17). For these, the results of ^210^Po and ^210^Pb activity concentrations in the whole specimens were calculated based on the activity concentration and biomass (caps and stipes) percentage share in the fruiting bodies (Table 1). Each analytical sample of boletes (4–5 g) had been spiked with 10 mBq of ^209^Po before radiochemical analysis as an internal tracer, and all prepared samples were digested using a concentrated solution (65%) of nitric acid (HNO_3_) [16]. The residues obtained were dissolved in 0.5M solution of HCl with added ascorbic acid. The activity concentration of ^210^Pb in analyzed samples was calculated indirectly via the activity measurement of its daughter ^210^Po. After at least six months of deposition time, the activities of the ingrown ^210^Po was measured in an alpha spectrometer (Canberra-Packard, USA). ^210^Pb activities measured in the studied boletes were calculated at their time of collection using the simplified equation for the daughter activity as a function of time [17]. The ^210^Po and ^210^Pb yield in the analyzed mushroom and soil samples ranged from 90 to 98%. The measurement results of ^210^Po and ^210^Pb activity concentrations were given with standard deviation (SD) calculated for 95% confidence intervals. The method’s accuracy was assessed using an IAEA reference material (IAEA-414) and participation in IAEA intercomparison exercises were estimated at better than 95%. Because of the abnormal distribution of radionuclides, non-parametric tests were used (*U*-test Mann–Whitney and *H*-test Kruskal–Wallis) to assess the significance of results, and the most important level achieved was quoted. Generally, the defined significance level was *p* = 0.05. The interpolation maps were prepared using QGIS software (QGIS Development Team) and results for the whole mushrooms.

An important aspect of chemical contaminants in biota is their uptake and distribution in the species. It has to be mentioned that the vegetative (main) body of basidiomycetes is the mycelium that is buried in the substrate, while the fruiting body (the mushroom) is an ephemeral reproductive organ used for dispersing spores into the surrounding space. Since the collection of a mycelium under natural forest conditions is generally discouraged by local customs (and regulation in some cases) because of the apparent potential damage to the habitat, it was not included in our study. Thus, to know approximately the distribution/localization of an element in a mushroom (sometimes the only cap is suitable or used for the culinary purpose) and to calculate its bioconcentration factor, a mushroom is separated into cap and stipe, which are examined individually. Therefore, it is possible to calculate, in the simplest mathematical way (no presentation of an equation/formula is necessary), the quotient of the occurrence (distribution) of an element within a specimen, expressed as the Q_C/S_ index (cap to stipe using normalized results for fully dehydrated materials).

The value of Q_C/S_ index > 1 (sometimes also called as distribution ratio, DR) shows that an element is preferentially accumulated in the caps [2,3,4,5,6,7,11,18,19]. The pattern of ^210^Po and ^210^Pb allocation in the morphological parts may change while ageing (maturing) [20], although mushrooms are generally not consumed when they reach this stage.

## 3. Results and Discussion

### 3.1. ^210^Po and ^210^Pb Activity Concentrations in Boletes

The activity concentrations determined in the bolete samples from Poland and Belarus showed a heterogeneous distribution of ^210^Po and ^210^Pb (Table 1). The activity concentrations (of ^210^Po and ^210^Pb, respectively) in whole mushrooms were in the range from 0.91 ± 0.10 Bq∙kg^−1^ db in Wysokie, to 4.47 ± 0.28 Bq∙kg^−1^ db in Osowa, and from 0.82 ± 0.09 Bq∙kg^−1^ db (Wysokie) to 5.82 ± 0.32 Bq∙kg^−1^ db (Elbląg), respectively. The results of ^210^Po and ^210^Pb activities in the Belarusian samples were similar to those in Poland (Table 1).

These activities (Table 1) were lower when compared to data reported for *Boletus* spp. studied in other countries. For example, ^210^Po and ^210^Pb activity concentrations in Finnish mushrooms ranged from 1.38 to 1174 ± 248 Bq∙kg^−1^ db; in German mushrooms, from 1.0 to 640 Bq∙kg^−1^ db; in Norwegian mushrooms, from 4.7 to 198 Bq∙kg^−1^ db; in Chinese mushrooms, from 1.66 to 308 Bq∙kg^−1^ db; while in other Polish species, the range was 0.23 to 36.4 Bq∙kg^−1^ db [12,21,22,23,24,25,26,27].

The statistical analysis showed a lack of significant differences in ^210^Po or ^210^Pb activity concentrations among the five bolete species (*H*-test Kruskal–Wallis *p*-value 0.33 for ^210^Po and 0.37 for ^210^Pb). There was also a lack of significant differences in the distribution of the nuclides between caps and stipes for each individual species and sampling point (*U*-test Mann–Whitney *p*-value 0.85 for ^210^Po and 0.59 for ^210^Pb). The bioconcentration in terrestrial species depends on the geochemical background and atmospheric fallout, but data for these parameters were not available for the boletus samples in the present study. The activities of ^210^Po and ^210^Pb in examined *B. edulis, B. pinophilus, B. reticulatus, B. luridus* and *B. impolitus* can be considered as low, suggesting that these species, similar to mushrooms of other genera from the *Boletaceae* family examined previously, namely the genus *Leccinum* and *Leccinellum*, do not strongly bioconcentrate ^210^Po and ^210^Pb [11,18,19]. However, again, this will depend to some extent on the background. Previously, Gwynn et al. have reported that differences in ^210^Po activity concentrations for individual specimens of the same mushroom species from the same stand were generally less than a factor of 3 in most cases [22].

**Figure 1:**
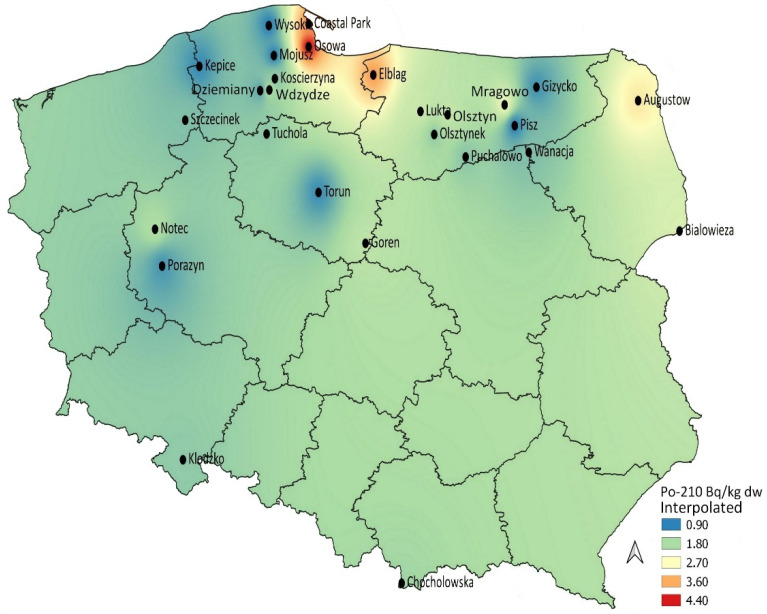
Interpolation map for ^210^Po activity concentrations in boletes.

**Figure 2:**
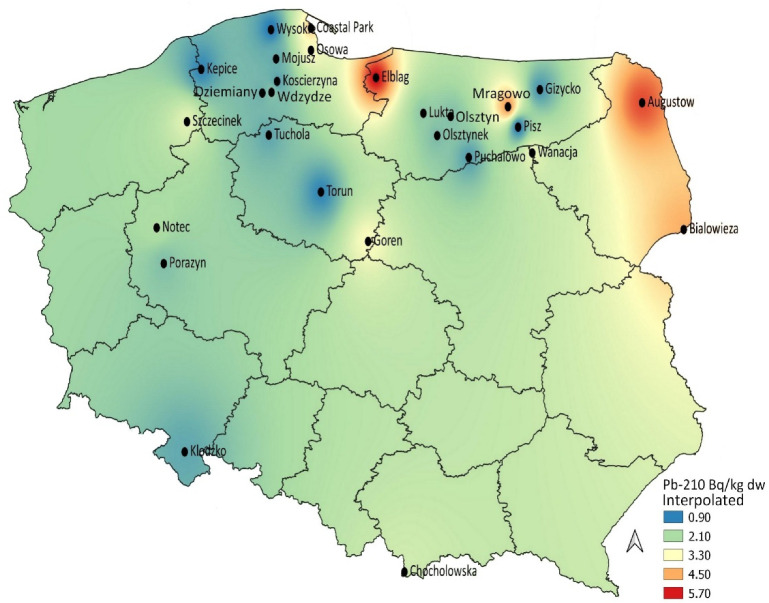
Interpolation map of ^210^Pb activity concentrations in boletes.

In an attempt to visualize the spatial occurrence of ^210^Po and ^210^Pb activities in five bolete species in Poland, the activities were mapped in Figure 1 and Figure 2. ^210^Po mushrooms from the northern and north-eastern regions of the country (Figure 1) appeared to be more contaminated, while the occurrence of ^210^Pb was more heterogeneous (Figure 2). Samples collected from northern and north-eastern regions were relatively more contaminated. The ^210^Pb spatial occurrence was similar to that noticed earlier for the Parasol Mushroom *Macrolepiota procera* [28], although activity concentrations were lower in boletes. This may be explained by a number of factors, such as the natural elemental occurrence in the soil bedrock, the depth of mycelium penetration in the substrate and also by the feeding behavior and/or nuclide enrichment in the organic matter of the soil (*Macrolepiota* are saprophytic and favor habitats that are rich in decaying organic matter). The boletes are mutual symbionts associated with a specific plant’s rhizosphere root system, and their interactions with trees, soil substrate and soil solution are more complicated [29]. As mentioned, the levels and distribution of both ^210^Pb and ^210^Po activity concentrations in the upper soil layers can be associated not only with the parent bedrock, but to some degree also with atmospheric fallout, where they are the result of the precipitation of radon decay products from the atmosphere and the level of ^210^Pb and ^210^Po contained in the top layer of soil can be correlated with the amount of atmospheric precipitation [10,30].

### 3.2. Distribution of ^210^Po and ^210^Pb within a Fruitbody

Analysis of the distribution of ^210^Po and ^210^Pb within the fruiting bodies of the boletes showed a wide range of Qc/s values, i.e., 0.60–1.67 for ^210^Po (mean value 1.00 ± 0.19 and median 1.07 ± 0.16) and 0.55–1.52 for ^210^Pb (mean 0.93 ± 0.18 and median 0.93 ± 0.13). There were no statistically significant differences between concentrations in the caps and stipes (*U*-test Mann–Whitney *p*-value 0.76) (Table 2). Generally, ^210^Po is more mobile in soil than ^210^Pb and easier bioconcentrated in fruiting bodies by some Basidiomycota [12]. Lead, including ^210^Pb, is known to be weakly bioconcentrated by numerous Basidiomycota studied so far, while stable lead is a notorious soil pollutant because of the legacy of historical industrial pollution and also current emissions from metal (lead, copper, zinc) smelters and other use [31,32,33,34,35,36]. Thus, sometimes, a relatively elevated concentration of stable Pb observed in mushrooms is due to the high degree of substrate soil pollution [31,32,34]. This does not apply to ^210^Pb that typically occurs at low activity concentration levels in mushrooms, as seen in this and other studies [27,35,36,37].

### 3.3. Annual Effective Radiation Doses for Adults

Based on the calculated ^210^Po and ^210^Pb content in dried boletes, the effective radiation doses were estimated (in 10 kg of an equivalent fresh mushroom portion) to identify their potential radiotoxicity to consumers (Table 3).

For adults, the effective dose conversion coefficients (dose per unit exposure) for ^210^Po and ^210^Pb ingestion that ICRP recommends for the calculation of equivalent and effective doses are 1.2 and 0.69 μSv∙Bq^−1^, respectively [38]. In the case of the bolete samples in this study, the consumption of whole mushrooms could lead to an effective ^210^Po radiation dose of 1.09 ± 0.12 to 5.37 ± 0.34 μSv∙kg^−1^ db with a corresponding dose of 0.57 ± 0.06 to 4.02 ± 0.22 μSv∙kg^−1^ db from ^210^Pb decay.

These calculated effective radiation dose values from the samples in this study are relatively low in comparison to other regular Polish food products, such as sea fish (24.6 µSv·y^−1^), dietary supplements (12 µSv·y^−1^), fresh red currants and potatoes (3 µSv·y^−1^), herbal teas (6.57 µSv·y^−1^), stimulants such as cigarettes (471 µSv·y^−1^) [16,39,40,41,42,43,44,45] or other mushrooms species such as *M. procera* (11.62 µSv·y^−1^) and *Leccinum* spp. (14.4 μSv∙kg^−1^ db) [11,18,19,28,46].

## 4. Conclusions

This study demonstrates that *B. edulis, B. pinophilus, B. reticulatus, B. luridus* and *B. impolitus* accumulate ^210^Po and ^210^Pb at different concentrations. The interpolation maps suggest a non-uniform spatial distribution of these nuclides based on their occurrence in common edible mushrooms. The occurrence indicates the geographical distribution of these nuclides across Poland, which also shows noticeable agreement with the natural radiological background. Morphologically, the ^210^Po and ^210^Pb quotients between cap and stipe (Q_c/s_) ranged from 0.55 to 1.67. Consumption of the analyzed mushrooms would result in a dose of 10 µSv∙kg^−1^ db in total, from both ^210^Po and ^210^Pb, which would not contribute significantly to the total annual effective radiation doses from ^210^Po and ^210^Pb intake from other sources for adult consumers. This suggests that consumption of these mushrooms is comparatively safe from the radiological protection point of view.

## Figures and Tables

**Table 1:** ^210^Po and ^210^Pb activity concentrations in boletes; ^a^ PM—Primeval Forest, ^b^ LP—Landscape Park; ^c^—^210^Po and ^210^Pb activities in whole fruiting bodies calculated based on levels in the caps and stipes and biomass share.

No	Sampling Site (Number of Individual Specimens)	Activity Concentration (Bq·kg^−1^ db)					
		Caps		Stipes		Whole Fruiting Bodies	
		^210^Po	^210^Pb	^210^Po	^210^Pb	^210^Po	^210^Pb
	Poland, *B. edulis*						
1	Kościerzyna (26)	-	-	-	-	1.63 ± 0.11	1.32 ± 0.04
2	Tuchola Pinewoods (6)	-	-	-	-	1.61 ± 0.10	1.42 ± 0.09
3	Toruńske Forest (15)	-	-	-	-	1.05 ± 0.08	0.99 ± 0.07
4	Łukta (24)	-	-	-	-	2.15 ± 0.11	1.86 ± 0.06
5	Puchałowo (15)	-	-	-	-	1.59 ± 0.08	1.32 ± 0.05
6	Osowa (15)	-	-	-	-	4.47 ± 0.28	3.27 ± 0.23
7	Olsztyn (19)	-	-	-	-	2.10 ± 0.20	1.74 ± 0.07
8	Augustów PF ^a^ (16)	-	-	-	-	2.91 ± 0.18	5.28 ± 0.30
9	Szczecinek (22)	-	-	-	-	1.53 ± 0.06	2.92 ± 0.15
10	Porażyn (13)	-	-	-	-	1.13 ± 0.08	1.74 ± 0.10
11	Mrągowo (15)	-	-	-	-	2.53 ± 0.13	4.76 ± 0.28
12	Chochołowska Dale (12)	-	-	-	-	1.75 ± 0.11	2.65 ± 0.11
13	Elbląg (21)	-	-	-	-	3.69 ± 0.14	5.82 ± 0.32
14	Mojusz (11)	-	-	-	-	0.92 ± 0.07	1.46 ± 0.09
15	Białowieża PF ^a^ (15)	-	-	-	-	2.31 ± 0.11	4.43 ± 0.27
16	Wanacja (15)	-	-	-	-	1.54 ± 0.09	3.02 ± 0.14
17	Goreń (15)	-	-	-	-	2.03 ± 0.09	3.29 ± 0.15
18	Piska Wilderness (15) ^c^	1.12 ± 0.08	0.94 ± 0.06	0.75 ± 0.06	0.80 ± 0.05	0.93 ± 0.52	0.87 ± 0.41
19	Giżycko (21) ^c^	1.15 ± 0.08	1.23 ± 0.09	1.04 ± 0.07	1.21 ± 0.06	1.10 ± 0.59	1.22 ± 0.60
20	Seacoast LP ^b^ (9) ^c^	1.76 ± 0.10	1.58 ± 0.06	2.82 ± 0.13	2.94 ± 0.07	2.30 ± 0.86	2.27 ± 0.48
21	Kłodzka Dale (10) ^c^	1.40 ± 0.09	1.22 ± 0.05	1.84 ± 0.13	1.62 ± 0.10	1.62 ± 0.80	1.42 ± 0.58
	*B. pinophilus*						
22	Piska Wilderness (15) ^c^	1.52 ± 0.08	1.59 ± 0.08	1.07 ± 0.06	1.13 ± 0.07	1.30 ± 0.39	1.36 ± 0.44
23	Noteć Forest (32) ^c^	2.38 ± 0.16	2.54 ± 0.14	1.42 ± 0.12	1.87 ± 0.12	1.89 ± 0.52	2.20 ± 0.47
24	Wdzydze LP ^b^ (26) ^c^	1.51 ± 0.11	1.30 ± 0.09	2.25 ± 0.13	2.36 ± 0.14	1.88 ± 0.69	1.83 ± 0.67
25	Dziemiany (14) ^c^	0.94 ± 0.08	1.03 ± 0.09	1.57 ± 0.09	1.96 ± 0.12	1.27 ± 0.27	1.53 ± 0.32
	*B. reticulatus*						
26	Seacoast LP ^b^ (15) ^c^	4.31 ± 0.24	4.54 ± 0.14	3.24 ± 0.23	3.25 ± 0.13	3.82 ± 1.17	3.95 ± 0.66
	*B. luridus*						
27	Wysokie (12) ^c^	0.94 ± 0.09	0.95 ± 0.08	0.86 ± 0.07	0.63 ± 0.09	0.91 ± 0.10	0.82 ± 0.09
28	Kępice (15) ^c^	1.04 ± 0.06	0.98 ± 0.08	0.97 ± 0.07	1.05 ± 0.09	1.02 ± 0.53	1.00 ± 0.68
	*B. impolitus*						
29	Olsztynek (15) ^c^	1.53 ± 0.11	1.72 ± 0.09	1.77 ± 0.13	1.66 ± 0.10	1.65 ± 0.68	1.69 ± 0.53
	Belarus, *B. reticulatus*						
30	Chojniki (38) ^c^	3.18 ± 0.13	1.80 ± 0.12	2.85 ± 0.16	2.46 ± 0.12	3.03 ± 0.77	2.11 ± 0.62
31	Borysów (34) ^c^	1.16 ± 0.07	1.33 ± 0.08	1.45 ± 0.09	1.63 ± 0.10	1.29 ± 0.39	1.46 ± 0.45

**Table 2:** Average values of the Q_c/s_ distribution for ^210^Po and ^210^Pb in boletes.

Sampling Site	Distribution (Cap/Stipe) (Q_c/s_)	
	^210^Po	^210^Pb
Poland, *B. edulis*		
Piska Wilderness	1.48 ± 0.10	1.18 ± 0.08
Giżycko	1.11 ± 0.11	1.01 ± 0.11
Seacoast Landscape Park	0.63 ± 0.16	0.54 ± 0.09
Kłodzka Dale	0.76 ± 0.16	0.75 ± 0.11
*B. pinophilus*		
Piska Wilderness	1.42 ± 0.10	1.41 ± 0.11
Notecka Forest	1.67 ± 0.21	1.36 ± 0.19
Wdzydze Landscape Park	0.67 ± 0.17	0.55 ± 0.17
Dziemiany	0.60 ± 0.13	0.53 ± 0.15
*B. reticulatus*		
Seacoast Landscape Park	1.33 ± 0.33	1.40 ± 0.19
*B. luridus*		
Wysokie	1.09 ± 0.12	1.52 ± 0.12
Kępice	1.07 ± 0.09	0.93 ± 0.12
*B. impolitus*		
Olsztynek	0.86 ± 0.17	1.04 ± 0.13
Belarus, *B. reticulatus*		
Chojniki (BY)	1.11 ± 0.20	0.73 ± 0.16
Borysów (BY)	0.80 ± 0.11	0.82 ± 0.13
Mean	1.00 ± 0.19	0.93 ± 0.18
Median	1.07 ± 0.16	0.93 ± 0.13

**Table 3:** Calculated average values of the effective radiation dose for adults from ^210^Po and ^210^Pb decay through *Boletus* spp. consumption.

Sampling Site	Effective Radiation Dose (μSv·kg^−1^ db)	
	^210^Po	^210^Pb
Poland, *B. edulis*		
Pomerania, Kościerzyna	1.95 ± 0.13	0.91 ± 0.02
Pomerania, Tuchola Pinewoods	1.93 ± 0.13	0.98 ± 0.07
Kujawy region, Toruńskie forests	1.27 ± 0.09	0.68 ± 0.05
Warmia, Łukta	2.58 ± 0.13	1.28 ± 0.04
Warmia, Puchałowo	1.91 ± 0.10	0.91 ± 0.03
Pomerania, Osowa	5.37 ± 0.34	2.26 ± 0.16
Warmia, Olsztyn	2.52 ± 0.24	1.20 ± 0.05
Podlasie, Augustów Primeval Forest	3.49 ± 0.22	3.64 ± 0.21
Pomerania, Szczecinek	1.84 ± 0.07	2.02 ± 0.10
Greater Poland, Porażyn	1.35 ± 0.09	1.20 ± 0.07
Masuria, Mrągowo	3.03 ± 0.15	3.28 ± 0.19
Tatra Mountains, Chochołowska Dale	2.10 ± 0.14	1.83 ± 0.08
Warmia, Elbląg	4.43 ± 0.17	4.02 ± 0.22
Pomerania, Mojusz	1.10 ± 0.08	1.01 ± 0.06
Podlasie, Białowieża Primeval Forest	2.78 ± 0.13	3.05 ± 0.18
Podlasie, Kurpiowska Forest, Wanacja	1.85 ± 0.11	2.09 ± 0.10
Kujawy region, Goreń	2.44 ± 0.10	2.27 ± 0.10
Masuria, Piska Wilderness	1.12 ± 0.63	0.60 ± 0.28
Masuria, Giżycko	1.32 ± 0.71	0.84 ± 0.41
Pomerania, Seacoast Landscape Park	2.76 ± 1.03	1.57 ± 0.33
Sudety Mountains, Kłodzka Dale	1.94 ± 0.96	0.98 ± 0.40
*B. pinophilus*		
Masuria, Piska Wilderness	1.56 ± 0.47	0.94 ± 0.30
Notecka Forests	2.26 ± 0.62	1.52 ± 0.33
Pomerania, Wdzydze Landscape Park	2.26 ± 0.83	1.26 ± 0.46
Pomerania, Dziemiany	1.53 ± 0.32	1.05 ± 0.22
*B. reticulatus*		
Pomerania, Seacoast Landscape Park	4.59 ± 1.41	2.72 ± 0.45
*B. luridus*		
Wysokie	1.09 ± 0.12	0.57 ± 0.06
Pomerania, Kępice	1.22 ± 0.63	0.69 ± 0.47
*B. impolitus*		
Warmia, Olsztynek	1.98 ± 0.81	1.17 ± 0.36
Belarus, *B. reticulatus*		
Gomel region, Chojniki	3.63 ± 0.92	1.46 ± 0.43
Minsk region, Borysów	1.55 ± 0.47	1.01 ± 0.31

## Data Availability

Data supporting reported results are available on request.
